# Establishment and Verification of Neural Network for Rapid and Accurate Cytological Examination of Four Types of Cerebrospinal Fluid Cells

**DOI:** 10.3389/fmed.2021.749146

**Published:** 2022-01-24

**Authors:** Luyue Jiang, Gang Niu, Yangyang Liu, Wenjin Yu, Heping Wu, Zhen Xie, Matthew Xinhu Ren, Yi Quan, Zhuangde Jiang, Gang Zhao, Wei Ren

**Affiliations:** ^1^Electronic Materials Research Laboratory, School of Electronic Science and Engineering, The International Joint Laboratory for Micro/Nano Manufacturing and Measurement Technology, Xi'an Jiaotong University, Xi'an, China; ^2^The College of Life Sciences and Medicine, Northwest University, Xi'an, China; ^3^Biology Program, Faculty of Science, The University of British Columbia, Vancouver, BC, Canada; ^4^School of Microelectronics, Xidian University, Xi'an, China; ^5^The State Key Laboratory for Manufacturing Systems Engineering, The International Joint Laboratory for Micro/Nano Manufacturing and Measurement Technology, Xi'an Jiaotong University, Xi'an, China

**Keywords:** neural network, white blood cell, cerebral spinal fluid, classification, clinical, image recognition

## Abstract

Fast and accurate cerebrospinal fluid cytology is the key to the diagnosis of many central nervous system diseases. However, in actual clinical work, cytological counting and classification of cerebrospinal fluid are often time-consuming and prone to human error. In this report, we have developed a deep neural network (DNN) for cell counting and classification of cerebrospinal fluid cytology. The May-Grünwald-Giemsa (MGG) stained image is annotated and input into the DNN network. The main cell types include lymphocytes, monocytes, neutrophils, and red blood cells. In clinical practice, the use of DNN is compared with the results of expert examinations in the professional cerebrospinal fluid room of a First-line 3A Hospital. The results show that the report produced by the DNN network is more accurate, with an accuracy of 95% and a reduction in turnaround time by 86%. This study shows the feasibility of applying DNN to clinical cerebrospinal fluid cytology.

## Introduction

The central nervous system (CNS) is one of the most crucial systems in the human body. One important aspect of the CNS is the cerebral spinal fluid (CSF), which is typically sterile and only contains around 1–5 white blood cells (WBCs) per microliter (μl) under normal conditions. Many neurological diseases cause changes in cerebrospinal fluid cytology, especially in infectious diseases of the nervous system. When perturbed by an infectious disease, the human body responds by increasing WBC population leading to an inflammation of the CNS, which leads to increased mortality and morbidity if not correctly diagnosed and properly treated. The global burden of CNS infections in 2016 was tabulated in a recent study (1) and estimated to be 9.4 million incidences with a mortality rate of 5% or 458,000 deaths annually. With such a high clinical priority and impact, there is always a need for improvement on the aspect of rapid diagnose for CNS infection.

The current diagnostic method for CNS infections consists of a series of tests, such as CSF test, culturing, and gram staining. In developing countries, the sensitivity of culturing and gram staining is low (2). CSF test is the most commonly used and includes several crucial key factors, such as cell counting, cell staining, and cell identification. Treatment usually begins at the onset of signs of CNS inflammation, immediately after the cell count and differential cell count become abnormal. This WBC identification is typically achieved with the May-Grünwald-Giemsa (MGG) staining of the CSF, which stains the nucleus and granules of the WBCs. In the case of one of the biggest hospitals in the northwestern region in China where this study is conducted, the hospital annually treats 120,000 outpatients with neurological diseases and among these, 4,000 patients are suspected of CNS infections (3). Because of this number, the hospital employs a large number of resources with an estimated 10 working hours per day dedicated just for CSF cell counting, cell staining, and cell identification alone.

Recent years have seen the boon of machine learning for analyzing large datasets and in particular, deep neural network (DNN) has been used to help to analyze and differentiate red blood cells (RBCs) and WBCs in whole blood (4–10). These studies imply different tactics, such as image segmentation, clustering, thresholding, local binary pattern, and edge detection (6). However, the initial implementation of these strategies for this application resulted in low clinical accuracies, thus accommodating a more generalized model, a generic object-detection neural network, such as region-based convolutional neural network (R-CNN), was explored and found to be more successful (11). To date, there have not been any studies for WBC differentiation in CSF using any machine-learning algorithms to the best of our knowledge.

In this study, the objective is to explore the feasibility of letting DNN to completely replace the currently employed manual labor leading to significant improvement in cell counting accuracy and cost savings. DNN is utilized in the differentiation of lymphocyte, monocyte, neutrophil, and erythrocytes for CNS inflammation diagnosis. To highlight how DNN accomplishes this, there are three main pillars presented in this study: (1) systematic validation of the DNN to confirm the similar quality of care to current standards, (2) analysis of accuracy and precision in automation, and (3) analysis of time savings if applied to the real case. The data reported in the present study are expected to greatly improve patient care when it comes to the diagnosis of infectious CNS diseases.

## Materials and Methods

### MGG Staining Procedure and OM Capture Details

Patients suspected of CNS inflammation had their CSF drawn from a typical lumbar puncture, where usually 10 ml of CSF was collected. The CSF was then split into two parts: (1) for cell count, 10 μl of CSF was dropped onto a hemocytometer and cells were counted, (2) based on the cell count, a proportional amount of CSF was used in the cytocentrifuge, and the cells were concentrated onto a microscope slide. MGG staining was done by first taking the microscope slide out and fixing them with acetone-formaldehyde. After they were fixed, the slides were stained with an MGG staining kit. The samples were then observed under a normal optical microscope (Leica DM2500) with the 20 × lens used first to get a general idea of the patient's condition. Additional 100 × images were subsequently taken when particular cells of interest were located; these 100 × images were the type sent to the DNN for training and testing. Afterward, the fixed microscope slides were preserved in a sample bank in case of future analysis.

### Preprocessing

All images were taken using an optical microscope (Leica DM2500) with the 100 × lens. Images are in the format of 8-bit JPEG. Each image was individually labeled with the type of each cell using an open-source software called Labellmg (12) by trained technicians with cell identification experience of 10 y. The Labellmg also helps to establish spatial locations of each cell by the function of the “user draw boxes”. Then, the saturation, the contrast, and the brightness of all images were randomly adjusted. All images were also randomly horizontally flipped.

### Training and Inference

Model training was performed in Python 3.6 and TensorFlow 1.14 using two NVIDIA 2080Ti 11 GB graphics processing units. Models were based on the Faster R-CNN architecture. The DNN software is a region-based convolution neural network (CNN) so it has great edge detection capability. It uses label mapping to separate labeled areas from the non-labeled background areas. Labeled images were split into two sections with a ratio of 9:1 and were separately put into the training and the testing folders, respectively. Model weights were initialized with weights pre-trained on the COCO database. Models were trained for 4-way classification (lymphocytes, monocytes, neutrophils, and RBCs). The RMSprop optimizer was used with a softmax loss and an exponential decay rate schedule with an initial learning rate of 0.001. Models were trained for 32,000 steps. The batch size was 4 and the Intersection over Union (IOU) threshold was 0.5. The model for each training episode was selected based on the PASCAL VOC detection metrics on the validation set. Predictions were averaged across all models and all cell images to produce a final prediction for each case. An external test set comprised of images from the rest of the dataset was used to evaluate the generalization performance of the model. Preprocessing scripts were written in Python to organize the data for utilization in TensorFlow. And the training was done until the loss function was saturated and observed *via* Tensorboard. Once the newly trained model was frozen, validations were done on the test image folder and compared with the ground truths of the trained technicians. After a reasonable accuracy was achieved, additional unlabeled images were evaluated with the frozen DNN model. For the training process of the DNN, 1,300 images, which include around 30,000 cells, were individually labeled and fed into the program.

## Results/Discussion

### Validating the DNN

**Figure 1:**
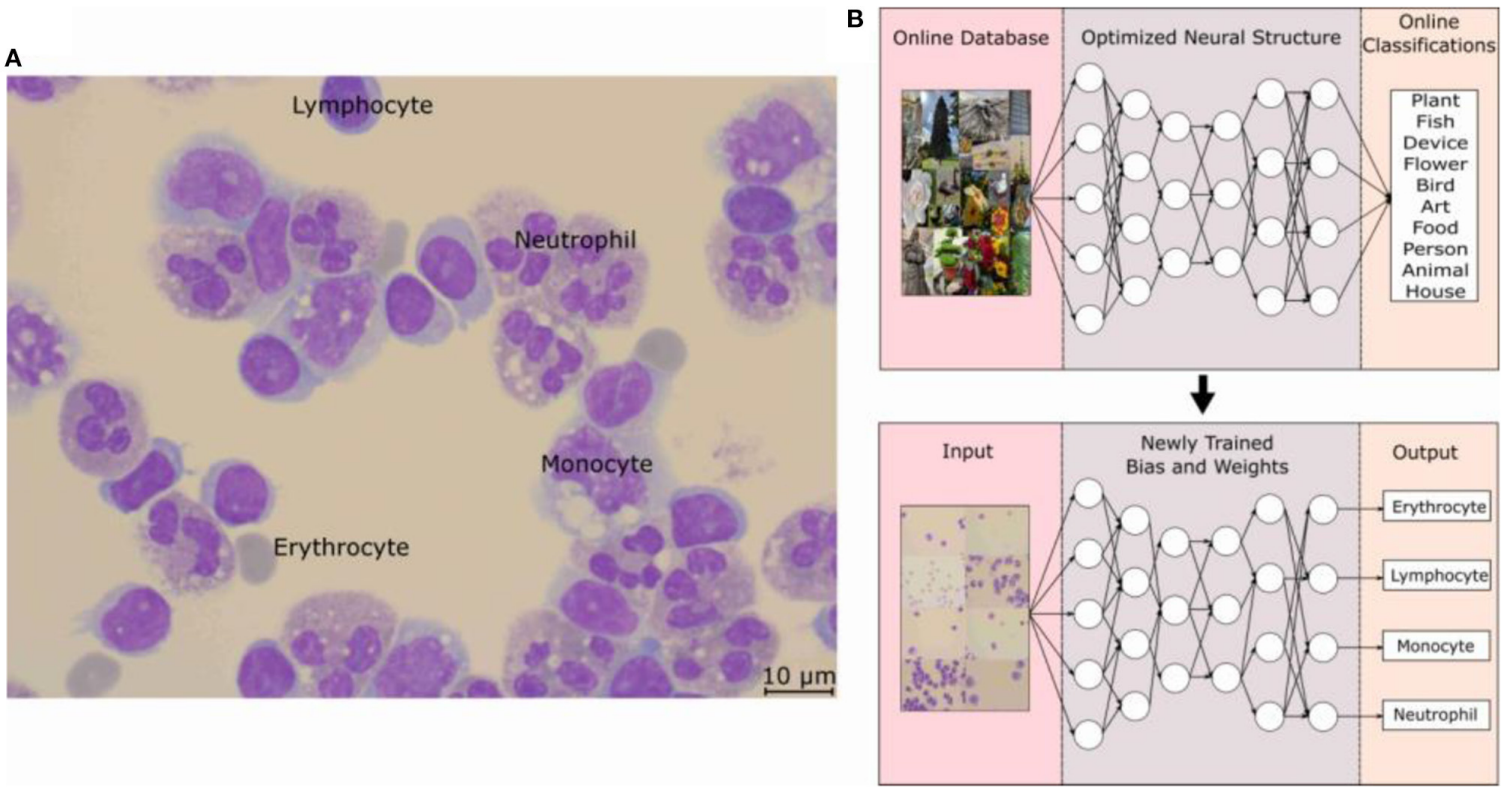
**(A)** Optical microscope image taken at 100 × with a scale bar of 10 μm. Cells were fixed and stained with MGG, which provides a light color to the cytoplasm of the cell and purple color to the lobes of the nucleus. Labels for the three WBCs and the RBCs can be seen in the picture. **(B)** Schematic of how the object-detection DNN model is trained to form its basic architecture. The model structure is Faster R-CNN with an initial learning rate of 0.001, a batch size of 4, and an IOU threshold of 0.5. The structure along with an online database was used to train the DNN model and then with the basic architecture, the weights and biases are optimized for the MGG-stained cell images of each classification. MGG, The May-Grünwald-Giemsa; DNN, deep neural network; RBC, red blood count; WBC, white blood cell.

The application of the DNN in this study is in the identification of the 4 main types of cells found in infectious CNS disease patients' CSF. The four main types of cells typically found are lymphocyte, monocyte, neutrophil, and erythrocytes. When a doctor suspects a CNS infection, the routine procedure of lumbar puncture is done and CSF is withdrawal from the patient, which will be stained for clear cell identification by the hospital technicians. The MGG staining provides a red acidic stain, a blue basic stain, and a purple color for cellular components (13, 14). This effectively gives the RBCs a dark gray or red-pink color, the WBCs a blue color with the lymphocyte a distinctive singular round purple nucleus, the monocyte with a large and bean-shaped purple nucleus, and finally the neutrophil with multi-lobed purple-colored nucleus (15). An example of MGG staining is shown in Figure 1A, where all four types of cells can be seen from CSF for one patient.

The DNN model employed for this study is based on an object-detection image-based neural network built on the TensorFow and pre-trained on the COCO dataset (16). The basics of a neural network can be considered as a repeating algorithm that classifies the importance of an input based on an activation function. An activation function is similar to the action potential of a human neuron cell, where a necessary stimulus causes the firing of the neuron, which is an all-or-nothing process. This is analogous to artificial neural networks where the activation function is a mathematical threshold value and once that is met, the result is similar to the firing of a human neuron. There are additional nuances to this mathematical equation with a coupling of weights and bias values, and the resultant firing is not a step function, but a specialized mathematical function containing in-between 0 and 1 activation values; an example is the sigmoid function. However, the main concepts translate to the idea that only the important characteristics of an image will be filtered through this activation function with each of these characteristics being represented as a neuron in one layer of the neural network. The addition of multiple layers gives rise to the non-linearly of a DNN and these features allow a DNN to recognize an image, similar to mimicking the image processing of a human brain. Coupled with the introduction of CNN, the processing requirement for image-based neural networks dropped significantly, paving the way for large advancements in the field (17). However, the detailed description and workings of each of these improvements are beyond the scope of this study, and a sample of this literature can be found in References. (11, 18, 19).

The application of the DNN to recognize WBCs and RBCs was made possible by first applying the pre-trained DNN to a database of optical microscope (OM) images labeled by doctors for each cell classification. The specifics of the Faster R-CNN model used can be found in this study (11, 19) and the training on the open-source image database, COCO by Microsoft (16), allowed for a DNN architecture to handle the complexities of the various cell types. As can be seen in Figure 1B, this pre-trained DNN model has already predetermined the number of layers, and neurons are needed for an optimal score of the COCO database and by carrying out a process of transfer learning (20), this model has re-trained itself by adjusting its weights and biases for MGG-stained cell images. The specific structure of the DNN model is Faster R-CNN, and an initial learning rate was 0.001, the batch size was 4, and the IOU threshold was 0.5.

**Figure 2:**
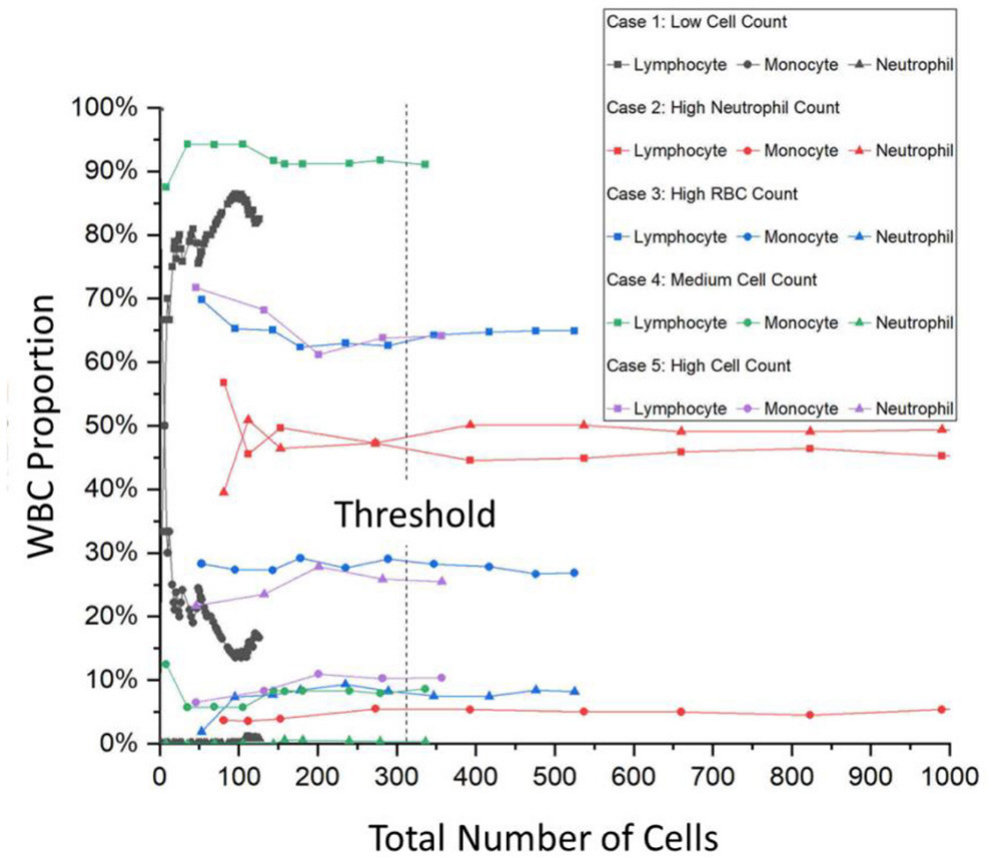
A trend graph of the number of cells per patient needed for certain patient condition examples. It can be seen that after a certain amount of cells, the percentage of the WBC type saturates thus determining the number of cells needed for a successful and accurate hospital report. This trend graph shows that on average, 315 cells are needed with even the onset of saturation starting at around 150 cells. WBC, white blood cells.

The typical hospital protocol in WBC type classification involves checking around 200 cells per patient. This is known as the cell classification step and it is one of the most time-consuming processes for the hospital. As can be seen in Supplementary Table 1, there are significant numbers of patients the hospital handles daily and as such, the hospital has the CSF Cytology Department to devote half-day daily to handle the suspected CSF samples. According to the hospital, the 200 cell minimum is an arbitrary standard set a while ago without much scientific basis but has not led to failure. As such, an objective study was also done to determine the minimum number of cells needed per patient and also to determine the minimum number of images needed to be taken per patient. Figure 2 shows the result of this focused study where only the three main WBC types are compared with the total number of cells identified per patient. For a typical hospital CSF cytology report, the doctors base their diagnosis on the percentage of these WBCs. The CSF Cytology Department has stored cytological smears of more than 100,000 patients in the last 10 y. Among these cytological smears diseases, 5 different cases can be categorized: (1) low WBC count (W = 0–4), (2) high neutrophil cell count, (3) high RBC count, (4) medium WBC count (W = 5–50), and 5) high WBC count (W ≥ 50). According to the characteristics of these types, we selected the corresponding cytological smears. Then we collected OM images of cells on each smear to analyze the threshold number of cells and the percentage of each cell type and subsequently to determine the required collecting cell number for each smear.

For Case 1, the low cell count typically means that the CSF of the patient is within the normal range and that the symptoms exhibited by the patient are from a different cause. However, Case 1 also has another difficulty where the entire cytospin sample contains typically <200 cells. As can be seen in Figure 2, the gray curves depict this, and the saturation of the curves is not met. For Cases 2–5, there are enough cells present and Figure 2 shows that saturation of the curves occurs after 315 cells are labeled. This number was calculated from an average of all the curves and from interpolations between data points after the minimum condition of saturation occurred. The onset of saturation can also be seen around 150 cells, but the error margin of 5% can be calculated.

**Figure 3:**
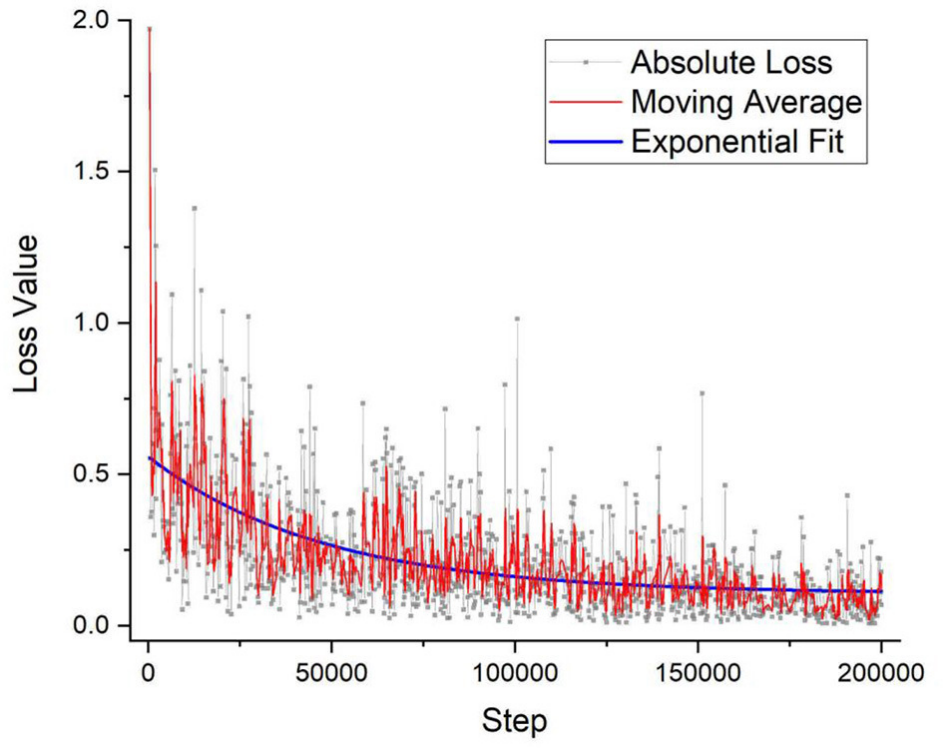
The DNN model's training accuracy shows its precision vs. the number of iteration steps. As can be seen that the graph takes on a 1/x, asymptotic relationship with saturation quickly established within the first few thousand steps. After 200,000 iterations, the precision % has not improved that much and the training of the model stopped, which took around 2 days of nonstop training. DNN, deep neural network.

For the training of the DNN, 100 × OM images were taken, and every cell in each image was labeled by a trained technician and cross-checked with specialized doctors. For the training process of the DNN, 1,300 images or around 30,000 cells were individually labeled and fed into the program. To verify the effectiveness of the training process, Figure 3 shows the loss value plotted against the number of iterations. The lower the value of the loss function indicates the more fully trained the DNN model. The loss function has become to an absolute limit of 0, which indicates that the model is perfectly trained. Generally, all DNN models are given trained values with a certain amount of noise, or in this case, a variety of images of different situations, so that the DNN can have the flexibility and not be over-fitted to a degree that it cannot identify images perfectly matching its initial training dataset. Figure 3 shows the output loss values in gray along with a moving average for a better visual representation of the graph. An exponential decay function is also fitted to highlight the saturation of the loss function. The training of this DNN took around 200,000 iterations and around 2.5 days. However, once a DNN is trained, it requires only around 7 s for an output.

### The Precision of the Neural Network

Besides merely relying on the loss function plot, a cross-check of the validation was performed to verify the accuracy of the DNN model. A certain portion of the image dataset was kept from training as the testing validation set, and the ratio amount chosen was 9:1. For comparison, four trained technicians were also arranged to label the same validation dataset, and then their results were compared with the DNN's prediction. Table 1 shows the labeling results of the validation dataset comparing the variations between the human labeling and the labeling of the DNN. The immediate takeaway is the confirmation that the multiple evaluation rounds of the DNN will produce the same result, however, that is not always the case. As can be seen in Supplementary Figure 1, there is a possibility for the DNN within the same validation round and with the same model version to produce two different image labeling outputs. In this case, the four validation rounds did not produce any variations. The other interesting factor comes from the human side with SD among the technicians producing large variability. However, such inaccuracy is suitable in the clinical setting where speed is more important and the WBC typing percentage can have a swing of ± 10% as the MGG cell classification report is only one of the many diagnostic tests typically done in series on a patient's CSF. This further shows the importance of implementing artificial intelligence (AI) in cell classification to improve the accuracy of the clinical results to reduce the reliance on subsequent tests in aiding the diagnosis of the doctor.

**Table 1:** The number of cells labeled in the validation dataset between human and AI for each cell type.

	**Erythrocyte**	**Lymphocyte**	**Monocyte**	**Neutrophil**
Human				
Person 1	13	66	67	41
Person 2	12	43	48	40
Person 3	20	77	70	43
Person 4	27	80	73	54
Human std dev	7	17	11	6
DNN				
Round 1	28	77	66	50
Round 2	28	77	66	50
Round 3	28	77	66	50
Round 4	28	77	66	50
AI Std Dev	0	0	0	0

**Figure 4:**
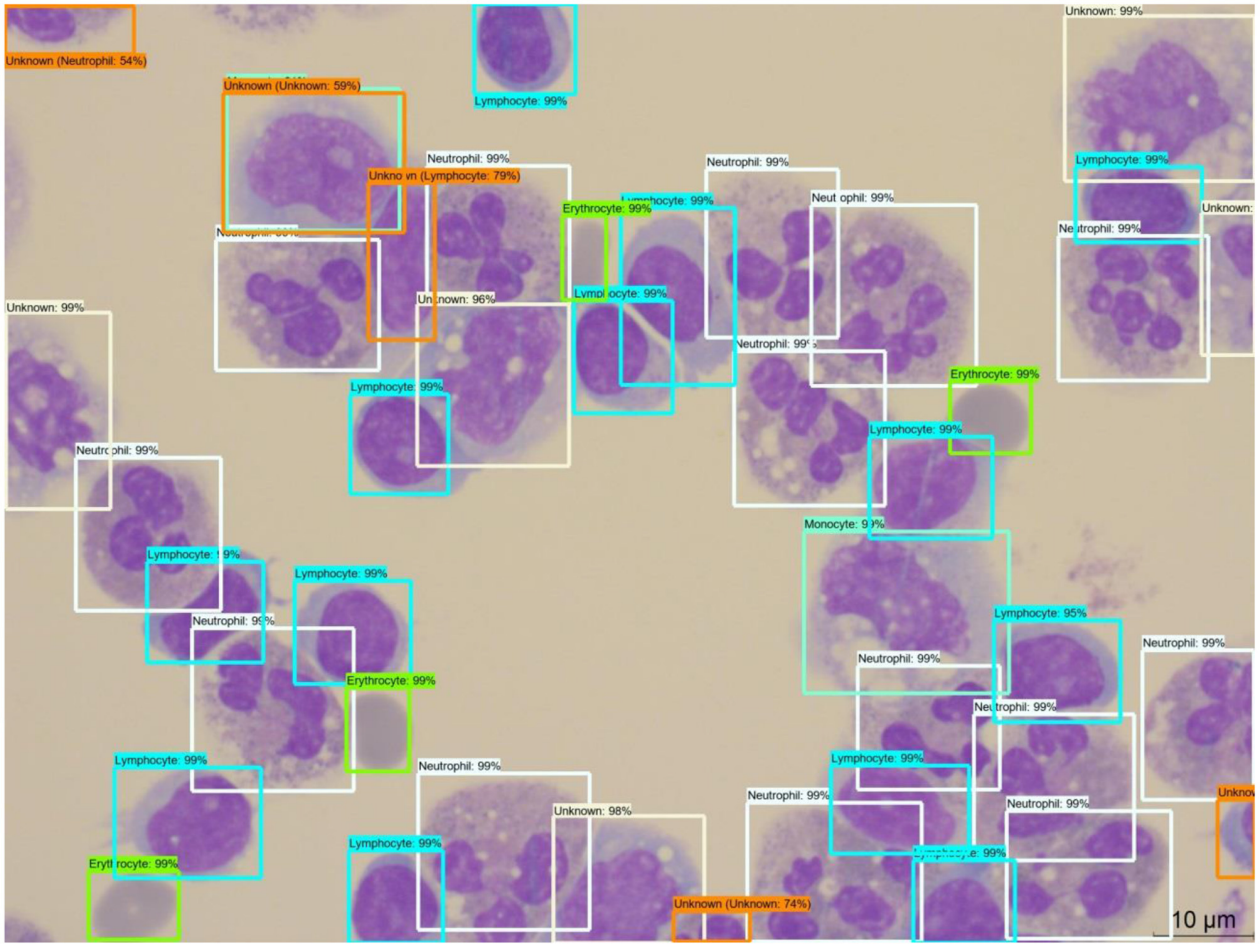
An example output of the DNN model with boxed labels along with the model's percentage prediction. One can see the predicted outputs of neutrophil, monocyte, lymphocyte, and erythrocyte with their respective colors along with the DNN model's percentage prediction. In addition, some cells are labeled with the “unknown” label tag (tan and orange boxes) when the prediction percentage is below 80% or when the shape of the cell indicates a possibility of a rare cell type (i.e., basophil, eosinophil, mitotic, etc.). DNN, deep neural network.

There are two outputs of the DNN program: (1) a labeled image with each DNN-recognized cell boxed with its prediction percentage, and (2) a report with the statistics of the recently run evaluation. An example of the output image can be seen in Figure 4 where the four major cell types are labeled by the DNN program. The program puts a predicted boxed area around the target cell and then gives each cell a classification prediction percentage. If that percentage falls under 80%, then the program will instead add another orange box over the original label and give it the label “unknown” so that a human technician can manually check the cell. Moreover, the cells that the program outputs the light brown boxes correspond to the “unknown” label, which is the more rare cell types (lymphoid, mitotic, basophil, etc.) and these will require the human technicians to check them as well. While the spatial location is innovative, it has not been widely used for common diagnosis reports, the percentage of WBC types is important for diagnosis, and the program calculates and outputs a statistical report of the three major WBC types.

### Accuracy of the Neural Network

To determine the effectiveness of the DNN in a real-world application setting, a blind test was performed and the comparison can be found in Table 2. During the blind test, the images were taken by operators without knowledge of the hospital report and given to a DNN operator, without any patient information except their ID number. The ID number is scrambled with the key being kept by a third party. From Table 2, the average differences show that the DNN model is fairly accurate when compared with the hospital report with the largest margin of error in cell classification with neutrophil and the largest patient variability with Patient #1. Overall, the DNN was able to handle the various infectious disease cases presented to it, they are as follows: (1) high neutrophil count, (2) high RBC count, (3) even distribution of WBC types, and (4) high lymphocyte count. The average accuracy of this DNN for these three WBC types is 95%. Compared to similar studies done on whole blood, our result is on similar levels of accuracy (6–8, 10). In addition, the average accuracy of this DNN is similar to these three WBC types of the same patient. Patient #2 provides a sample with a high neutrophil count. For Patient #2, the average error of this DNN for these three WBC types is minimal, i.e., only ~1.3%. For Patient #3 with a close amount of three WBC types, the average error of this DNN for these three WBC types is ~6%. In addition, for Patient #4 with a high lymphocyte count, the average error of this DNN for these three WBC types is ~2.3%. For Patients #2–4, the error of this DNN for monocytes is minimal compared to those of lymphocytes and neutrophils. However, for Patient #1, neutrophils and monocytes showed large recognition errors. Upon closer inspection of the data discrepancy for Patient #1, it was found that the DNN had not previously encountered abnormal neutrophil images during its training phase. These abnormal neutrophil pictures had the individual nuclei lobes clustered together into a similar shape of the monocyte nuclei producing a false negative result; an example of this can be seen in Supplementary Figure 2. These misclassifications led to the uneven monocyte/neutrophil percentage and thusly incorrect report. To better apply the DNN for future clinical situations, the training regime will have more of an emphasis on the number of patients trained rather than the number of cells trained for each cell classification to account for the complex clinical patient situations.

**Table 2:** Blind testing results of the DNN vs. the Hospital Diagnosis Report.

	**% Lymphocyte**	**% Monocyte**	**% Neutrophil**
Hospital Technician			
Patient 1 (ID# 190931)	5	3	92
Patient 2 (ID# 191155)	1	7	91
Patient 3 (ID# 191172)	18	23	59
Patient 4 (ID# 191158)	87	6	7
DNN			
Patient 1 (ID# 190931)	7	18	75
Patient 2 (ID# 191155)	4	7	90
Patient 3 (ID# 191172)	27	20	53
Patient 4 (ID# 191158)	89	8	3
Comparison Between Human vs AI			
Patient 1 (ID# 190931)	2	16	16
Patient 2 (ID# 191155)	3	0	1
Patient 3 (ID# 191172)	9	3	6
Patient 4 (ID# 191158)	2	2	3
Average Difference	4	5	7

### Time-Saving Potential

One of the main advantages of using the DNN program to replace the mundane task of cell type labeling is the time savings for the doctors so that their attention can be more focused on other tasks. To quantify these time savings, a short survey was conducted during a working week to estimate the time committed on each patient and daily basis. An example of the complete survey can be found in Supplementary Table 1, Table 3 shows the time required by the hospital personnel for the two time-saving procedures that the DNN can contribute: 1) cell classification and 2) report writing. As can be seen in Table 3, the DNN can save around 16 min per patient and around 4 h per day; this amounts to a doctor time reduction of 86% daily. The DNN time was calculated from the validation dataset and extrapolated with an average number of patients from the short survey. The minimum number of cells per patient, extrapolated from Figure 2, and the average number of cells per image were also factors used. In addition, the DNN processing time required per image was also found to be independent of the number of cells present, with processing time slowing down as heat became more difficult to dissipate from the machine.

**Table 3:** The time-saving potential when compared between the DNN and hospital technician.

	**Average time per day (mins)**	**Average time per patient (mins)**	**%**
Hospital technician			
Cell classification	211 ± 25.3	13.4 ± 0.86	N/A
Report writing	70 ± 13.4	4.4 ± 0.20	N/A
Total time	281 ± 38.5	17.8 ± 0.92	N/A
DNN			
Cell classification	34 ± 4.5	2.2 ± 0.04	N/A
Report writing	3 ± 0.3	0.2 ± 0.00	N/A
Total time	37 ± 4.8	2.4 ± 0.04	N/A
Time saved	243 ± 38.8	15.5 ± 0.92	86 ± 4

*DNN, deep neural network*.

## Conclusion

This study presents a pioneering application of image-based DNNs to patient samples in a clinical setting. Image analysis of MGG-stained patient samples is done for CSF cytology. By applying neural network technology to the clinical space of cell-type classification, a significant saving in time has been achieved. The daily saving in the time spent counting cells of hospital technicians is estimated to be approximately 86 ± 4%. DNN further rendered more consistent analyses capability against the large variability common to human classification analyses. Blind tests result in an average accuracy of 95% among the three WBC types, with the addendum being that the accuracy of the program can always be improved further with additional training from a wider variety of patients. This report clearly demonstrates the promise of DNN in clinical practices pertaining to infectious diseases of the CNSs.

## Data Availability Statement

The raw data supporting the conclusions of this article will be made available by the authors, without undue reservation.

## Ethics Statement

This article does not contain any studies with human participants or animals performed by any of the authors. No modifications were done to this procedure, patients received routine care, and everything was approved in accordance with the local Ethics Committee. The MGG databank follows protocols in accordance with ethical standards of the Fourth Military Medical University and with the 1964 Helsinki declaration and its later amendments or comparable ethical standards. Images in the dataset do not contain any identifying information and patients willing consented to have their stained cell images stored in this MGG databank.

## Author Contributions

LJ, GN, ZJ, GZ, and WR contributed to conception and design of the study. LJ collected the pictures of cells and wrote the first draft of the manuscript. LJ, YQ, and WY performed the statistical analysis. LJ, MR, YL, and HW designed the DNN model. ZX proofread and edited the manuscript. All authors contributed to manuscript revision, read, and approved the submitted version.

## Funding

Key R&D Program of Shaanxi Province of China (2020GY-271), the 111 Project of China (B14040), the National Natural Science Foundation of China (Program No. 81671185), the Natural Science Basic Research Program of Shaanxi (Program No. 2019JQ-251), the Hospital-level project of Xi'an International Medical Center (Program No. 2020ZD007).

## Conflict of Interest

The authors declare that the research was conducted in the absence of any commercial or financial relationships that could be construed as a potential conflict of interest.

## Publisher's Note

All claims expressed in this article are solely those of the authors and do not necessarily represent those of their affiliated organizations, or those of the publisher, the editors and the reviewers. Any product that may be evaluated in this article, or claim that may be made by its manufacturer, is not guaranteed or endorsed by the publisher.
