# Supplementary material for: Establishment and Verification of Neural Network for Rapid and Accurate Cytological Examination of Four Types of Cerebrospinal Fluid Cells
Source: Front Med (Lausanne). 2022 Jan 24;8:749146. doi: 10.3389/fmed.2021.749146 (PMC8818991; doi:10.3389/fmed.2021.749146)
Supplement: Supplementary file 1 [file Data_Sheet_1.pdf]

Supplementary Information

Establishment and verification of neural network for rapid and accurate cytological examination of four types of cerebrospinal fluid cells

Luyue Jiang[1], Gang Niu[1*], Yangyang Liu[1], Wenjin Yu[2], Heping Wu[1], Zhen Xie[2], Matthew Xinhu Ren[3], Yi Quan[1, 4], Zhuangde Jiang[5], Gang Zhao[2*], Wei Ren[1*]

[1] Electronic Materials Research Laboratory, School of Electronic Science and Engineering & the International Joint Laboratory for Micro/Nano Manufacturing and Measurement Technology, Xi'an Jiaotong University, Xi'an 710049, China

gangniu@xjtu.edu.cn ; wren@xjtu.edu.cn

[2] The College of Life Sciences and Medicine, Northwest University, Xi'an, Shaanxi, 710069, P.R. China

zhaogang@nwu.edu.cn

[3] Biology Program, Faculty of Science, The University of British Columbia, Vancouver, BC, V6T 1Z4, Canada

[4] School of Microelectronics, Xidian University, Xi’an, 710071, China

[5] The State Key Laboratory for Manufacturing Systems Engineering & The International Joint Laboratory for Micro/Nano Manufacturing and Measurement Technology， Xi'an Jiaotong University, Xi'an 710049, China

1. Inconsistencies with DNN

While it is typically assumed that computer models are consistent in their outputs and that this is a trivial statement, this is not always the case for a DNN. Part of the training of a DNN is the inherent addition of noisy images to allow for flexibility in the decision-making of the model. If a model is trained too well, this leaves the model with the inability to determine answers that are not exactly within its database. An example of such DNN indecision can be seen in **S. Fig. 1** where the same images were processed in the same evaluation round with the same model, however, two outputs were presented.

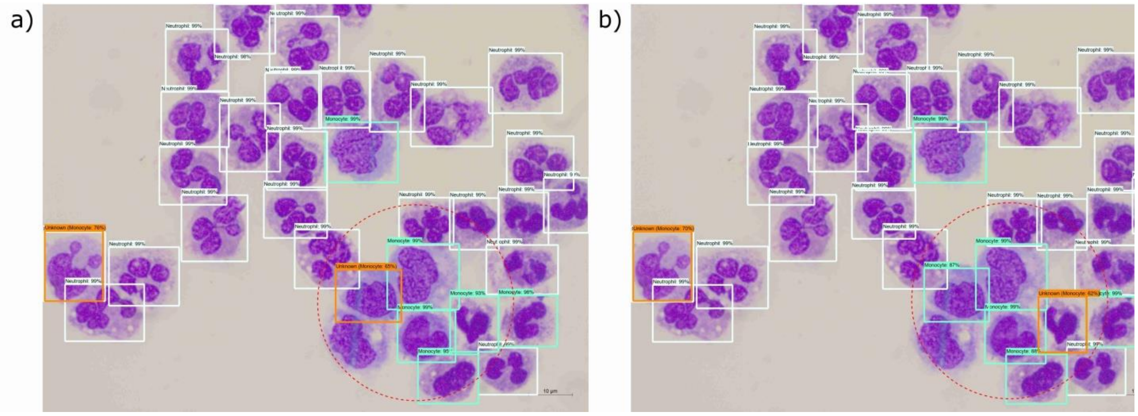

S. Fig. 1 – Two DNN output images showing the inconsistency of a model in determining cell classifications. These two images happened to be run during the same evaluation round and same model, yet the model still produced two different results. The red circle in a) and b) shows the difficulty in the model in identifying the two different monocyte cells as noted by the orange "unknown" label.

2. Typical CSF Cytology Lab Time Data

Over the period of 1 week (4 working days), the time required for each step of the MGG staining protocol is recorded. The purpose of this was to get an estimate of the time needed per patient and the amount of resources that the hospital needs to dedicate for each patient. Based upon this, it was seen that the DNN can help in the cell classification step and save a large portion of the doctor's time.

S. Table 1 – A collection of the time needed per major step in the MGG staining process

日期（date）	序号(serial number)	编号(report number)	本院(Xijing Hospital)	外院 (Other Hospital)	住院号(Patient ID)	细胞计数情况 (cell count results)		细胞计数(Cell Counting)				细胞离心沉淀(centrifugal slide sedimentation method)				MGG染色(MGG Staining)				细胞分类计数(Cell classification count)				报告(Report)			
						R	W	开始时间(start time)		结束时间(end time)	间隔 (time interval)	开始时间(start time)		结束时间(end time)	间隔 (time interval)	开始时间(start time)		结束时间(end time)	间隔 (time interval)	开始时间(start time)		结束时间 (end time)	间隔 (time interval)	开始时间(start time)		结束时间 (end time)	间隔 (time interval)
	1	190818	1		896110	0	3																		8:50/14:00	8:51/14:02	3min
	2	190819	1		60810712	39	3																		8:51/14:02	8:53/14:04	4min
	3	190238	1		808975	0	1																		8:55/14:04	8:55/14:06	4min
	4	190820	1		F66901	1	1														9:09	9:19	10min+1min		8:55/14:06	8:56/14:07	2min
	5	190774	1		F65295	0	0														9:19	9:29	10min+1min		8:56/14:07	8:57/14:09	3min
	6	190239	1		F31911	0	0														9:29	9:40	11min+1min		9:17/14:10	9:19/14:12	4min
	7	190284	1		F48356	193	24														9:40	9:54	14min+1min		9:19/14:14	9:20/14:16	3min
	8	180890	1		E98891	52	4														9:54	10:08	14min+1min		9:20/14:16	9:21/14:18	3min
	9	190821	1		60817048	402	5														10:07	10:22	15min+1min		9:22/14:18	9:24/14:21	5min
5/6/2019	10	190822		1	19207361	0	0													5/7/2019	10:08	10:12	4min+1min		9:24/14:23	9:25/14:26	5min
	11	190823	1		81029721	60	41														10:10	10:27	17min		9:25/14:27	9:26/14:36	7min
	12	190799	1		F66802	24	484														9:54	10:06	12min+1min		9:26/14:36	9:27/14:40	5min
	13	182271		1	346676	0	3														9:46	9:53	7min+1min		9:27/14:41	9:28/14:42	2min
	14	170849		1	263620	0	1														9:38	9:46	8min+1min		9:28/14:42	9:29/14:44	3min
	15	190824		1	1079990	11	0														10:14	10:25	11min+1min		9:35/14:44	9:36/14:46	3min
	16	181584		1	334758	42	0														10:25	10:34	9min+1min		9:36/14:46	9:37/14:47	2min
	17	151997		1	212170	10	0														11:36	11:48	12min+1min		9:37/14:47	9:38/14:49	3min
	18	190825		1	577511	0	22														10:23	10:39	16min+1min		9:38/14:50	9:40/14:52	4min
	19	190826	1		F67604P	58	486														11:12	11:46	34min+1min		9:40/15:07	9:41/15:09	3min
	20	190741	1		F625229	1	2														10:38	10:50	12min+1min		9:41/14:52	9:43/14:56	6min
	1	151592	1		C75365	2	2		13:51	13:52	1min				10min				10min		8:30	8:45	15min+1min		10:32	10:35	3min
	2	190827		1	O737230	0	2		13:52	13:53	1min				10min				10min		8:40	8:55	15min		10:35	10:37	2min
	3	190828		1	50997	40	508		13:53	13:56	3min				10min				10min		9:11	9:34	23min+1min		10:39	10:43	4min
	4	190829	1		7-67295	24	0		13:56	13:57	1min				10min				10min		9:35	9:45	10min+1min		10:46	10:52	6min
	5	170823	1		E20484	2	3		13:57	13:58	1min				10min				10min		9:45	9:51	6min+1min		10:52	10:56	4min
	6	190830		1	577619	0	6		14:01	14:02	1min		14:16	14:26	10min		15:00	15:10	10min		8:58	9:11	12min		10:57	11:00	3min
5/7/2019	7	162271	1		D72078	46	2	5/7/2019	13:58	13:59	1min	5/7/2019			10min	5/7/2019			10min	5/8/2019	9:11:00	9:25:00	13min	5/8/2019	11:00	11:04	4min
	8	190831		1	1917111	28	5		14:03	14:04	1min				10min				10min		9:25	9:34	9min		11:04	11:08	4min
	9	181774		1	557825	9	1		13:59	14:00	1min				10min				10min		9:40	9:47	7min		11:08	11:10	2min
	10	190832		1		0	0		14:02	14:03	1min				10min				10min		9:47	10:00	13min		11:10	11:12	2min
	11	190833	1		D80885	0	1		14:04	14:05	1min				10min				10min		9:51	10:05	9min+1min		11:12	11:15	3min
	12	190834		1	19207728		0		15:01	15:02	1min		15:10	15:20	10min		15:30	15:40	10min		10:00	10:13	13min+1min		11:15	11:18	3min
	13	181866		1	19208235	0	6		15:06	15:07	1min				10min				10min		10:07	10:20	13min		11:18	11:20	2min
	1	171329	1		D74068	0	0		13:47	13:48	1min				15min				10min		8:38	8:45	7min				6min
	2	190801	1		1800559112	11	79		13:48	13:51	3min		14:25	14:40	15min		14:44	14:54	10min		9:45	9:57	14min+1min				6min
	3	190747		1		0	4		13:51	13:52	1min				15min				10min		9:10	9:25	15min+1min				6min
	4	190441	1		F57072	0	3		13:53	13:54	1min			14:47	22min		14:47	14:57	10min		8:43	9:10	27min+1min				6min
	5	190835				0	2		13:54	13:55	1min				15min		14:48	14:58	10min		9:25	9:35	10min				6min
	6	190836	1		F67765	0	0		13:55	13:56	1min		14:30	14:45	15min				10min		9:40	9:52	12min				6min
	7	190837	1		F67800	0	2		13:56	13:57	1min				15min		14:45	14:55	10min		9:55	10:05	10min				6min
5/8/2019	8	190838	1		F67047	0	1	5/8/2019	13:57	13:58	1min	5/8/2019		14:55	25min	5/8/2019	15:04	15:14	10min	5/9/2019	8:45	9:05	20min	5/9/2019	13:47	13:50	6min
	9	190839	1		F67699	0	0		13:58	13:59	1min		15:03	15:20	17min		15:20	15:30	10min		9:57	10:05	8min+1min				6min
	10	190840	1		F67407	0	4		13:59	14:00	1min				17min				10min		10:05	10:20	15min+1min				6min
	11	190841		1	1917832	0	2		14:04	14:07	3min		14:18	14:37	19min		14:41	14:51	10min		10:26	10:34	8min+1min				6min
	12	190842		1	1064172	44	0		14:07	14:08	1min		15:05	15:15	12min		15:15	15:25	10min		10:34	10:50	16min+1min				6min
	13	190843		1		2	2		14:08	14:10	2min			15:20	17min		15:20	15:30	10min		10:15	10:30	15min				6min
	14	190844		1		12	6		14:13	14:14	1min		15:00	15:15	15min		15:17	15:27	10min		9:58	10:04	6min+1min				6min
	15	190845		1	D35726	368	2		15:00	15:03	3min		15:24	15:35	11min		15:35	15:45	10min		9:35	9:43	8min+1min				6min
	16	190761	1		F69940	8	2		15:04	15:06	2min		15:00	15:15	15min		15:17	15:27	10min		9:26	9:34	8min+1min				6min
	1	190510		1	O36733	0	0		9:07	9:08	1min		9:11	9:20	9min		9:25	9:35	10min	5/9/2019	14:53	15:07	14min+1min		10:35	10:38	3min
	2	190846	1		F66770	2950	490		9:08	9:13	5min				9min				10min		15:08	15:55	47min+1min		10:47	10:58	6min
	3	190079	1		F36404	0	0		13:49	13:50	1min			14:08	8min		14:09	14:19	10min		8:40	8:55	15min+1min		10:38	10:45	7min
	4	190847		1	19208456	8	0		13:57	13:58	1min		14:00	14:25	25min		14:26	8:38	10min		9:52	10:07	15min+1min				5.5min
	5	190848		1	315536	0	1		13:50	13:51	1min			14:10	10min		14:15	14:25	10min		10:07	10:20	13min+1min				5.5min

3. Incorrect labeling by the DNN on Patient 1 (ID# 190931)

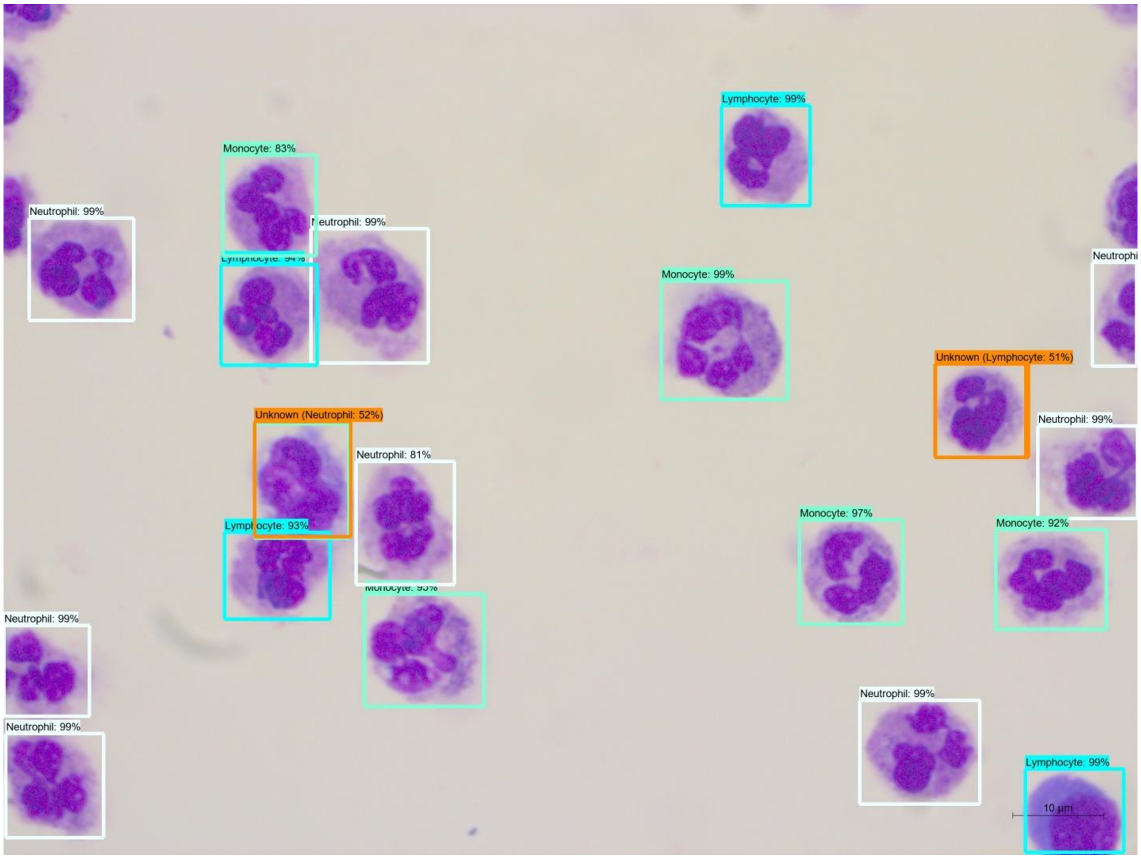

S. Fig. 2 – DNN output of the blind test with Patient 1 (ID# 190931). The mislabeling of the neutrophils can be seen in this image with the only true lymphocyte cell classification in the bottom right; the rest of the image should be all neutrophil classifications but the various granulate aggregation confuses the DNN to label these cells as lymphocyte or monocyte instead of neutrophil.

4. Cerebrospinal fluid cytology report of a patient

Check No. xxx

Cerebrospinal fluid cytology report of the Department of Neurology

Unit sending inspection samples: Xijing Hospital
Department: Neurology **Hospital-bed Number:** xx
Data sending inspection samples: 2018—01—09

Name: xxx **Sex:** Male **Age:** 50 **AD:** xxx
Clinical diagnosis: Central nervous system infection RBC count: 1436 /mm^3
CSF appearance: Colorless and transparent Cryptococcus count: 0 /mm^3
WBC count: 3 /mm^3

WBC classification:

Lymphocytes	92	%	(184)	Large lymphocytes		%	( )
Monocytes	4.5	%	(9)	Large lymphoid cells		%	( )
Phagocytes	0.5	%	(1)	Neutrophils	3	%	(6)
Exfoliated cells		%	( )	Eosinophils		%	( )
Other cells		%	( )	Basophils		%	( )
Plasma cells		%	( )	Mitotic cells		%	( )

Attached

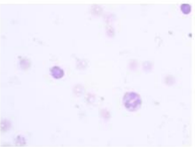

Fig. 1 (10 x 100)

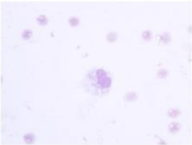

Fig. 2 (10 x 100)

Fig. 1: Optical microscope image of lymphocytes and neytrophils;
Fig. 2: Optical microscope image of phagocytes. Phagocytic substance was hemosiderin.

Diagnosis and Suggestions: The WBC count was lower than that on December 18, and no significant change in cytological classification was observed.
Reporter: xxx Report data: 2018 –01一10
This report is only responsible for the specimen.

S. Fig. 3 – Cerebrospinal fluid cytology report of the department of neurology.
